# Learning channel-selective and aberrance repressed correlation filter with memory model for unmanned aerial vehicle object tracking

**DOI:** 10.3389/fnins.2022.1080521

**Published:** 2023-01-10

**Authors:** Jianjie Cui, Jingwei Wu, Liangyu Zhao

**Affiliations:** ^1^School of Aerospace Engineering, Beijing Institute of Technology, Beijing, China; ^2^The Second Academy of CASIC, Beijing, China

**Keywords:** unmanned aerial vehicle, object tracking, discriminative correlation filter, channel regularization, aberrance repressed, historical memory

## Abstract

To ensure that computers can accomplish specific tasks intelligently and autonomously, it is common to introduce more knowledge into artificial intelligence (AI) technology as prior information, by imitating the structure and mindset of the human brain. Currently, unmanned aerial vehicle (UAV) tracking plays an important role in military and civilian fields. However, robust and accurate UAV tracking remains a demanding task, due to limited computing capability, unanticipated object appearance variations, and a volatile environment. In this paper, inspired by the memory mechanism and cognitive process in the human brain, and considering the computing resources of the platform, a novel tracking method based on Discriminative Correlation Filter (DCF) based trackers and memory model is proposed, by introducing dynamic feature-channel weight and aberrance repressed regularization into the loss function, and by adding an additional historical model retrieval module. Specifically, the feature-channel weight integrated into the spatial regularization (SR) enables the filter to select features. The aberrance repressed regularization provides potential interference information to the tracker and is advantageous in suppressing the aberrances caused by both background clutter and appearance changes of the target. By optimizing the aforementioned two jointly, the proposed tracker could restrain the potential distractors, and train a robust filter simultaneously by focusing on more reliable features. Furthermore, the overall loss function could be optimized with the Alternative Direction Method of Multipliers (ADMM) method, thereby improving the calculation efficiency of the algorithm. Meanwhile, with the historical model retrieval module, the tracker is encouraged to adopt some historical models of past video frames to update the tracker, and it is also incentivized to make full use of the historical information to construct a more reliable target appearance representation. By evaluating the method on two challenging UAV benchmarks, the results prove that this tracker shows superior performance compared with most other advanced tracking algorithms.

## 1. Introduction

The thinking ability endowed by the brain is fundamental. Due to its existence, human beings are more intelligent than animals. It is also the premise that humans have the capability to conduct scientific research (Kuroda et al., 2022). People rely on their brains to recognize the world, learn knowledge, and summarize rules. Their brains also allow them to use memory systems to store the information generated when experiencing different events (Atkinson and Shiffrin, 1968; Cornelio et al., 2022). In turn, the information serves as prior knowledge, helping people in dealing with similar problems better and adapting to new complex scenes faster. The core of artificial intelligence (AI) is to enable the machine to complete specific tasks independently through learning and using prior information (Connor et al., 2022; Foksinska et al., 2022; Nofallah et al., 2022; Pfeifer et al., 2022; Wang et al., 2022). The original scientific research mainly adopts the following two methods. The first is to mathematically represent the law (i.e. prior information) that people summarize when perceiving things, and then use mathematical expressions and logical frameworks to construct modules and methods for computers, just like teachers teach students what they know. The second is to build a variety of artificial neural networks based on the neural structure of the human brain and then use large-scale data to train and fit the network (Deng et al., 2022; Liu et al., 2022), aiming to enable the computer to automatically learn the characteristics of various things from the data itself, just like the students read books and learn by themselves. Although scientists have put a lot of effort into the research and utilization of the human brain, it is still a difficult task to determine how to endow computers with more and better prior knowledge through algorithms.

This paper mainly concentrates on visual object tracking on the UAV platform, which plays an important role in the field of computer vision, and is widely used in many tasks, such as collision avoidance (Baca et al., 2018), traffic monitoring (Elloumi et al., 2018), military surveillance (Shao et al., 2019), and aerial cinematography (Gschwindt et al., 2019). By adopting this technology, it aims to predict the precise status of the target in a video sequence captured by an onboard camera only with the information given in the first frame (Han et al., 2022). Over the past few years, a lot of effort has been put into the tracking field. However, it is still a challenging task to design a robust and efficient tracker, when considering the various complex UAV tracking scenarios, e.g., occlusion, change of viewpoint, and limited power capacity.

In the past decade, the research on visual object tracking mainly adopted the two methods below, namely the discriminative correlation filter (DCF)-based method and the Siamese-based method. The Siamese-based method (Bertinetto et al., 2016b; Li et al., 2018a; Wang et al., 2019; Voigtlaender et al., 2020; Javed et al., 2022) aims at the offline learning of the similarity measurement function between image patches, by maximizing the distance between the target and the background patches while minimizing the distance between the different image patches belonging to the same target. Such a method consists of two identical subchannels that are used to process the target template and the current frame search area, respectively. The target location is determined by computing the partial similarity between the target template and each location in the search area. Moreover, the Siamese-based method uses neural network architecture and numerous training data to obtain excellent feature extraction capability, so it needs to occupy a large number of computing resources in the tracking process. DCF-based methods are based on the correlation theory in the field of signal processing, and it computes the correlation between different image patches by convolution. Such a method usually adopts the hand-crafted features carefully designed with prior information and aims at training a correlation filter online in the region around the target by minimizing a least squares loss. Due to the convolution theorem, DCF-based methods can track objects at hundreds of frames per second (FPS) with only one CPU. Considering that the computing resources of the UAV platform are very limited, and the speed is a key issue in addition to the tracking performance, this paper mainly concentrates on target tracking based on DCF methods.

The development history of the DCF-based method is the process by which people integrated more and better prior information into the tracking framework. As people add their understanding of tracking tasks as regular constraints to the loss function (Mueller et al., 2017; Han et al., 2019b), the trained correlation filter becomes more and more discriminative and robust. Mosse (Bolme et al., 2010), as the originator of correlation filtering, deemed target tracking as a problem of binary classification, and trained the filter by randomly sampling a fixed number of background samples as negative samples. This greatly limits its discriminative power. To effectively increase the number of training samples, which was critical to the performance of the trained classifier, KCF (Henriques et al., 2014) introduced the circulant matrix into the tracking framework and obtained a large number of virtual negative samples by circularly shifting the target samples. The cyclic shifting greatly increased the training samples and caused boundary effects that seriously limited the improvement of tracking performance simultaneously. To mitigate the boundary effect, SRDCF (Danelljan et al., 2015) added the SR term into the loss function, aiming at penalizing the non-zero value near the template boundaries. BACF (Kiani Galoogahi et al., 2017) generated lots of real background samples, by expanding the search area and introducing a binary mask for middle elements cropping. To solve the scale change of the target, DSST (Danelljan et al., 2014a) introduced an independent scale filter, in addition to the classical correlation filter used for locating, as well as SAMF (Li and Zhu, 2014) sampled multiscale images, thereby building image pyramids. For the improvement of the feature representation, CN (Danelljan et al., 2014b) brought in color features, while ECO (Danelljan et al., 2017) added depth features obtained from off-line training of the neural network. STRCF (Li et al., 2018b) brought in additional temporal constraints to the SRDCF to limit the variation of the filter in consecutive frames. This effectively reduced the risk of filter degradation in case of sudden large appearance variations. SAT (Han et al., 2019a) advocated a kurtosis-based updating scheme to guarantee a high-confidence template updating. ASRCF (Dai et al., 2019) realized the adaptive suppression of clutter in different regions by regarding the SR term, introduced in SRDCF, as a variable. MUSTer (Hong et al., 2015) built the short-term and long-term memory stores, thereby processing the target appearance memories. Autotrack (Li et al., 2020) reformulated the loss function by introducing the change of response maps into temporal regularization (TR) and SR terms, thereby realizing adaptive adjustment. Regardless of the great progress in DCF-based tracking methods, there are still some issues to solve. (1) Most original trackers treat the features of different dimensions equally. Features of different dimensions play different roles in tracking different scenarios and different kinds of targets. The tracker is easily biased by similar interference due to ignorance of the feature channel information. (2) Most original trackers have insufficient ability to suppress potential interference. Most of the original methods merely utilize the same and fixed bowl-shaped SR term centered on the target, aiming at giving more weight to the background area for suppression. Additional suppression is not applied to the potential interference according to the actual tracking situation, thus leading to limited anti-aberrance capability. (3) Most original trackers do not effectively use historical information. Most of the original methods updated the filter with a constant update rate, thereby causing the waste of historical information and the risk of filter degradation. Historical information is one of the most important factors in the tracking process and should be efficiently used to enhance the discriminant capacity of the tracker.

The brain can perceive the interference information in the background, independently select the optimal features to describe the target, and use historical memory to achieve an accurate target location in the current frame. When considering the above, a UAV tracking algorithm with repressed dynamic aberrance, a channel selective correlation filter, and a historical model retrieval module is proposed to solve the aforementioned problems. Moreover, by formulating the dynamic feature channel weight and the aberrance repressed regularization into the integral loss function, the tracking algorithm is built, thereby enabling the filter to highlight valuable features in the channel domain and using response maps to sense and suppress background interference in advance. Meanwhile, the model retrieval module, by imitating brain memory realizes the adaptive update of the tracker. This paper has the main contributions as follows.

i) A novel tracking method, that integrates the aberrance repressed regularization and dynamic feature channel weight into the loss function of the DCF framework, is proposed. For joint modeling of the two factors, the tracker obtains the ability to screen target features based on actual background interference and learns more differentiated target appearance representation. Thus, the loss function could be solved in very few iterations by employing an efficient ADMM algorithm.

ii) A model retrieval module is employed which can realize the adaptive update of the tracker by saving the history filters. This module can also enhance the tracker's learning of the appearance of the trusted targets with historical information and reduce the pollution of unreliable samples for the tracker.

iii) By giving the experimental validation conducted on two public UAV datasets, the effectiveness of this method is demonstrated.

## 2. Proposed methodologies

### 2.1. Revisted autotrack

In this section, the baseline Autotrack of this tracker shall be revised.

Most original trackers, based on the discriminative correlation filters (DCF), attempt to add a variety of regularization terms such as spatial regularization (SR) and temporal regularization (TR), thereby improving the discrimination ability to target and background. Such regularization terms are usually predefined fixed parameters, so flexibility and adaptability are lacking in cluttered and challenging scenarios. To realize automatic adjustment of the hyper-parameters of the SR and TR terms during tracking, Autotrack constructs them with the response maps obtained during detection. Specifically, Autotrack introduces the partial response variation Λ to the SR parameter $\tilde{u}$, and the global response variation ∥**Λ**∥_2_ to the reference value $\tilde{\theta }$ of the coefficient of the TR term. The partial response variation Λ is defined as the variation of response maps between two continuous frames, with the Equation as below.


(1) $$\begin{array}{l} \Lambda =\frac{R_{t}\left[\psi_{\Delta }\right]-R_{t-1}}{R_{t-1}} \end{array}$$

Where, ***R*** refers to the response map calculated in the detection phase. [ψ_Δ_] represents the shift operator which makes the response peaks in response maps of two continuous frames coincide with each other. As for Autotrack, the integral objective loss function is shown below:


(2) $$\begin{array}{l} E\left(H_{t},\theta_{t}\right)=\frac{1}{2}{∥y-\overset{K}{\underset{k=1}{\sum }}x_{t}^{k}⊛h_{t}^{k}∥}_{2}^{2}+\frac{1}{2}\overset{K}{\underset{k=1}{\sum }}{∥\tilde{u}⊙h_{t}^{k}∥}_{2}^{2}\\ \;+\frac{\theta_{t}}{2}\overset{K}{\underset{k=1}{\sum }}{∥h_{t}^{k}-h_{t-1}^{k}∥}_{2}^{2}+\frac{1}{2}{∥\theta_{t}-\tilde{\theta }∥}_{2}^{2}\\ \text{ s.t. }\tilde{u}=P^{⊤}\delta log\left(\Lambda +1\right)+u\\ \;\tilde{\theta }=\frac{\zeta }{1+log\left(\nu {∥\Lambda ∥}_{2}+1\right)},∥\Lambda {∥}_{2}\leq \phi \end{array}$$

Where, $X_{t}=\left[x_{t}^{1},x_{t}^{2},...,x_{t}^{K}\right]$ and $H_{t}=\left[h_{t}^{1},h_{t}^{2},...,h_{t}^{K}\right]$ represent the trained filter and the extracted target feature matrix at t frame, respectively. K is the total number of feature channels. $x_{t}^{k}\in R^{T\times T}$ indicates the sample feature vector with length *T* in frame *t* in k channel and *y* ∈ ***R***^*T*×*T*^ is the desired corresponding label set in the Gaussian shape. $\tilde{u}$ and θ_*t*_ represent the coefficients of SR and TR, respectively. $\tilde{\theta }$ is the reference value of θ_*t*_ used for measuring the change in the tracking response map between two continuous frames. **P**^T^ ∈ *R*^*T*×*T*^ is a binary matrix, used in cropping the central elements of the training sample ***X***_*t*_. δ is a constant that can be used in balancing the weights of partial response variations. ***u*** represents a fixed bowl-shaped matrix of SR which is identical to the STRCF tracker. ⊛ and ⊙ represent the convolution operation and the elemental multiplication, respectively. $∥{∥}_{2}^{2}$ is the Euclidean norm.

SR and TR, constructed by response maps variation, enable the trained filter in Autotrack to adjust automatically while flying and be more adaptable to different scenarios. Although this method has achieved outstanding performance, it does have two limitations. a) This method uses the response map generated by the filter in the previous frame, rather than the learned filter in this frame, thus leading to insufficient suppression of interference. Sudden changes in response maps give important information regarding the similarity of the current object and the appearance model and reveal potential aberrances. The tracker should reduce the learning of irrelevant objects according to the changes during the training phase. b) The weight of each feature channel is equivalent. Different channels describe the objects in different dimensions. There may be many similar features between the target and the background, which are useless or even have a negative effect on the discriminatory ability of trackers. Thus, the filter selects partial distinctive features based on the actual situation for training and updating.

### 2.2. Loss function construction

To solve the above problems and enhance the discrimination ability and anti-interference ability of the tracker, the weight of the feature channel and aberrance suppression are introduced together to restrain the filter. Specifically, feature channel weight, which is treated as an optimization variable, updates simultaneously with the filter. Also, the variation of two continuous response maps, as an aberrance suppression regularization, is integrated into the training process. The loss function is shown below.


(3) $$\begin{array}{l} E\left(H_{t},\theta_{t},\upsilon_{t}\right)=\frac{1}{2}{∥y-\overset{K}{\underset{k=1}{\sum }}x_{t}^{k}⊛h_{t}^{k}∥}_{2}^{2}+\frac{1}{2}\overset{K}{\underset{k=1}{\sum }}{∥\upsilon_{t}^{k}\tilde{u}⊙h_{t}^{k}∥}_{2}^{2}\\ \;+\frac{\lambda_{1}}{2}\overset{K}{\underset{k=1}{\sum }}{∥\upsilon_{t}^{k}-\upsilon_{0}^{k}∥}_{2}^{2}\\ \;+\frac{\theta_{t}}{2}\overset{K}{\underset{k=1}{\sum }}{∥h_{t}^{k}-h_{t-1}^{k}∥}_{2}^{2}+\frac{1}{2}{∥\theta_{t}-\tilde{\theta }∥}_{2}^{2}\\ \;+\frac{\lambda_{2}}{2}{∥Q_{t-1}-\overset{K}{\underset{k=1}{\sum }}x_{t}^{k}⊛h_{t}^{k}∥}_{2}^{2} \end{array}$$

Where $\upsilon_{t}^{k}$ is the weight coefficient of feature channel k at t frame. It should be noted that $\upsilon_{t}^{k}$ is not a fixed parameter, but a variable that changes with the target appearance during the tracking. The constant $\upsilon_{0}^{k}$ is regarded as the reference of $\upsilon_{t}^{k}$, which represent the advance distributions of targets in the different feature channels. $\upsilon_{0}^{k}$ is set to 1, thereby ensuring that each feature channel has the same weight in the initial state. ***Q***_*t*−1_ refers to the response map generated from the previous frame, and is equivalent to $\overset{K}{\underset{k=1}{\sum }}x_{t-1}^{k}⊛h_{t-1}^{k}$. Thus, it can be treated as a constant signal during the optimization stage. λ_1_, and λ_2_ are parameters that control model overfitting.

Equation 3 consists of six items that can be divided into four parts. The first part constitutes the first item, the regression term. The second part, including the second and third items, is the SR integrated with channel selection. The third part, consisting of the fourth and fifth items, is the TR borrowed from Autotrack. The fourth part, made up of the last item, is the regularization term, aiming at restricting and counteracting the aberrances created by the background information. For the introduction of channel weight $\upsilon_{t}^{k}$, the feature sifting of the filter is realized in the channel domain by mitigating the impact of features having no relation to the targets and by excluding needless information. By introducing aberrance repressed regularization, which gives greater penalties for interference, the ability of the tracker to identify the aberrance in the background, and suppress the subsequent changes of response maps on the basis of the baseline, is further improved. The fusion of these two factors enables the filter to find the aberrance in time, and utilize the best features, thereby maximizing the differentiation between the target and background.

### 2.3. Optimization

As observed from Equation 3, the optimization of the overall loss function involves the complex correlation operation between matrices. Therefore, to reduce computational complexity, and reduce sufficient computing efficiency, the Parseval theorem is used to convert complex correlation operations into simple elemental multiplication operations and move the loss function from the time domain to the Fourier domain as $E\left(H_{t},{\hat{G}}_{t},\theta_{t},\upsilon_{t}\right)$. Besides, the constraint parameter ${\hat{g}}_{t}^{k}=\sqrt{T}FP^{\text{T}}h_{t}^{k}$ is used in constituting the Augmented Lagrangian function $L\left(H_{t},\hat{G_{t}},\upsilon ,\theta_{t},\hat{M_{t}}\right)$ as follows:


(4) $$\begin{array}{l} L\left(H_{t},\hat{G_{t}},\upsilon ,\theta_{t},\hat{M_{t}}\right)=E\left(H_{t},{\hat{G}}_{t},\theta_{t},\upsilon_{t}\right)\\ \;+\overset{K}{\underset{k=1}{\sum }}\left({\hat{g}}_{t}^{k}-\sqrt{T}FP^{\text{T}}h_{t}^{k}\right){\hat{m}}_{t}^{k}\\ \;+\frac{\mu }{2}\overset{K}{\underset{k=1}{\sum }}{∥{\hat{g}}_{t}^{k}-\sqrt{T}FP^{\text{T}}h_{t}^{k}∥}_{2}^{2}\\ \;E\left(H_{t},{\hat{G}}_{t},\theta_{t},\upsilon_{t}\right)=\frac{1}{2}{∥\hat{y}-\overset{K}{\underset{k=1}{\sum }}{\hat{x}}_{t}^{k}⊙{\hat{g}}_{t}^{k}∥}_{2}^{2}\\ \;+\frac{1}{2}\overset{K}{\underset{k=1}{\sum }}{∥\upsilon_{t}^{k}\tilde{u}⊙h_{t}^{k}∥}_{2}^{2}+\frac{\theta_{t}}{2}\overset{K}{\underset{k=1}{\sum }}{∥{\hat{g}}_{t}^{k}-{\hat{g}}_{t-1}^{k}∥}_{2}^{2}\\ \;+\frac{1}{2}{∥\theta_{t}-\tilde{\theta }∥}_{2}^{2}+\frac{\lambda_{1}}{2}\overset{K}{\underset{k=1}{\sum }}{∥\upsilon_{t}^{k}-\upsilon_{0}^{k}∥}_{2}^{2}\\ \;+\frac{\lambda_{2}}{2}{∥{\hat{Q}}_{t-1}-\overset{K}{\underset{k=1}{\sum }}{\hat{x}}_{t}^{k}⊙{\hat{g}}_{t}^{k}∥}_{2}^{2} \end{array}$$

Where symbol ˆ represents the discrete Fourier transformation (DFT), for example, $\hat{y}=\sqrt{N}Fy$ and ***F*** called the Fourier matrix is the orthonormal *N* × *N* matrix of complex basis vectors. ***m*** refers to the Lagrangian multiplier, and **μ** represents the penalty parameter. For simplification, $\hat{G_{t}}=\left[{\hat{g}}_{t}^{1},{\hat{g}}_{t}^{2},{\hat{g}}_{t}^{3},...,{\hat{g}}_{t}^{K}\right]$ and $\hat{M_{t}}=\left[{\hat{m}}_{t}^{1},{\hat{m}}_{t}^{2},{\hat{m}}_{t}^{3},...,{\hat{m}}_{t}^{K}\right]$ are defined. By assigning $\hat{s_{t}^{k}}=\frac{1}{\mu }\hat{m_{t}^{k}}$ the optimization of Equation (4) is equivalent to solving equation (5).


(5) $$\begin{array}{l} L\left(H_{t},{Ĝ}_{t},\upsilon ,\theta_{t},{Ŝ}_{t}\right)=E\left(H_{t},{Ĝ}_{t},\upsilon ,\theta_{t}\right)\\ \;+\frac{\mu }{2}\overset{K}{\underset{k=1}{\sum }}{∥{ĝ}_{t}^{k}-\sqrt{T}FP^{\text{T}}h_{t}^{k}+{ŝ}_{t}^{k}∥}_{2}^{2} \end{array}$$

Considering the complexity of the above-mentioned function, the alternative direction method of multipliers (ADMM) (Lin et al., 2010) is applied to speed up the calculation. Specifically, the function of optimization can be divided into a few sub-problems to be solved iteratively. During the solution of every subproblem, only one variable is contained to be optimized, while the others are regarded as fixed constants temporarily. In this way, each subproblem and its relevant closed-form solution can be given in detail below.

**Subproblem** for ${\hat{G}}_{t}$: By giving ***H***_*t*_, **υ**, θ_*t*_, ***Ŝ***_***t***_, the optimal ${\hat{G}}_{t}^{*}$ could be obtained by solving the optimization problem:


(6) $$\begin{array}{l} {\hat{G}}_{t}^{*}=\underset{{\hat{G}}_{t}^{*}}{\arg\min}\{\frac{1}{2}{∥\hat{y}-\overset{K}{\underset{k=1}{\sum }}{\hat{x}}_{t}^{k}⊙{\hat{g}}_{t}^{k}∥}_{2}^{2}+\frac{\theta_{t}}{2}\overset{K}{\underset{k=1}{\sum }}{∥{\hat{g}}_{t}^{k}-{\hat{g}}_{t-1}^{k}∥}_{2}^{2}\\ \;+\frac{\lambda_{2}}{2}{∥{\hat{Q}}_{t-1}-\overset{K}{\underset{k=1}{\sum }}{\hat{x}}_{t}^{k}⊙{\hat{g}}_{t}^{k}∥}_{2}^{2}\\ \;+\frac{\mu }{2}\overset{K}{\underset{k=1}{\sum }}{∥{ĝ}_{t}^{k}-\sqrt{T}FP^{\text{T}}h_{t}^{k}+{ŝ}_{t}^{k}∥}_{2}^{2}\} \end{array}$$

However, it is still very difficult to solve Equation 6 directly, because this subproblem containing ${\hat{X}}_{k}{\hat{g}}_{k}$ shows a high computation complexity and needs multiple iterations in ADMM. Fortunately, ${\hat{X}}_{k}$ is sparse, which means that each element of $\hat{y}$$\left(\hat{y}\left(n\right),n=1,2,\ldots ,N\right)$ is merely related to ${\hat{x}}_{k}\left(n\right)=\left[{\hat{x}}_{k}{\left(n\right)}^{1},{\hat{x}}_{k}{\left(n\right)}^{2},\ldots ,{\hat{x}}_{k}{\left(n\right)}^{D}\right]$ and ${\hat{g}}_{k}\left(n\right)=\left[conj\left({\hat{g}}_{k}{\left(n\right)}^{1}\right),conj\left({\hat{g}}_{k}{\left(n\right)}^{2}\right),...,conj\left({\hat{g}}_{k}{\left(n\right)}^{D}\right)\right]$, where *conj*() refers to the complex conjugate operation. Thus, this subproblem can be divided into *N* simpler problems across *K* channels as follows.


(7) Γj*(G^t)=argminΓj(G^t){∥y^j-Γj(X^t)⊤Γj(G^t)∥22     +μ∥Γj(G^t)+Γj(s^t)-Γj(TFP⊤Ht)∥22     +θt∥Γj(G^t)-Γj(G^t-1)∥22     +λ22∥Q^t-1-Γj(X^t)⊤Γj(G^t)∥22}

Where, $\Gamma_{j}\left({\hat{G}}_{t}\right)\in C^{(}K\times 1)$ indicates the vector including all *K* channel value of ${\hat{G}}_{t}$ on pixel *j*(*j* = 1, 2, …, *N*). By introducing the Sherman-Morrison formula ${\left(uv^{H}+A\right)}^{-1}=A^{-1}-\frac{A^{-1}uv^{H}A^{-1}}{v^{H}A^{-1}u+1}$, the inverse operation in the derivation can be further simplified and accelerated.Then, the closed-form solution of this subproblem can be obtained as follows.


(8) $$\begin{array}{l} \Gamma_{j}^{*}\left({\hat{G}}_{t}\right)=\frac{1}{\mu +\theta_{t}}\left(\text{I}-\frac{\left(1+\lambda_{2}\right)\Gamma_{j}\left({\hat{\text{X}}}_{t}\right)\Gamma_{j}{\left({\hat{\text{X}}}_{t}\right)}^{⊤}}{\theta_{t}+\mu +\left(1+\lambda_{2}\right)\Gamma_{j}{\left({\hat{\text{X}}}_{t}\right)}^{⊤}\Gamma_{j}\left({\hat{\text{X}}}_{t}\right)}\right)\rho \end{array}$$

Where **ρ** is merely an intermediate variable for simple representation and $\rho =\Gamma_{j}\left({\hat{\text{X}}}_{t}\right){\hat{\text{y}}}_{j}+\theta_{t}\Gamma_{j}\left({\hat{G}}_{t-1}\right)-\mu \Gamma_{j}\left({\hat{S}}_{t}\right)+\mu \Gamma_{j}\left(\sqrt{T}\text{F}{\text{P}}^{⊤}H_{t}\right)+\lambda_{1}\Gamma_{j}\left({\hat{\text{X}}}_{t}\right){\hat{Q}}_{t-1}$

**Subproblem** for ***H***_*t*_: By fixing ${\hat{G}}_{t}$,**υ**,θ_*t*_,***Ŝ***_***t***_, ***H***_*t*_ can be solved with the equation below:


(9) $$\begin{array}{l} h_{t}^{k*}=\underset{h_{t}^{k}}{\arg\min}\left\{\frac{1}{2}{∥\upsilon_{t}^{k}\tilde{u}⊙h_{t}^{k}∥}_{2}^{2}+\frac{\mu }{2}{∥{\hat{g}}_{t}^{k}-\sqrt{T}F{\text{P}}^{\text{T}}h_{t}^{k}+{\hat{s}}_{t}^{k}∥}_{2}^{2}\right\}\\ \;=\frac{\mu T\text{p}⊙\left(s_{t}^{k}+g_{t}^{k}\right)}{\lambda_{1}\left(\upsilon_{t}^{k}\tilde{u}⊙\upsilon_{t}^{k}\tilde{u}\right)+\mu T\text{p}} \end{array}$$

Where, $\text{p}={\left[{\text{P}}_{11},{\text{P}}_{22},\ldots ,{\text{P}}_{TT}\right]}^{⊤}$ represents the column vector, that composed of the diagonal elements of **P**. As observed in Equation 9, the computational cost on $h_{t}^{k*}$ solution is very low, because it only involves the element-wise operation and an inverse fast Fourier transform.

**Subproblem** for θ_*t*_: By treating ${\hat{G}}_{t}$,**υ**,***H***_*t*_,***Ŝ***_***t***_ as constants, the optimal θ_*t*_ can be obtained by solving the problem of optimization below:


(10) $$\begin{array}{l} \theta_{t}^{*}=\underset{\theta_{t}}{\arg\min}\left\{\frac{\theta_{t}}{2}\overset{K}{\underset{k=1}{\sum }}{∥{\hat{g}}_{t}^{k}-{\hat{g}}_{t-1}^{k}∥}_{2}^{2}+\frac{1}{2}{∥\theta_{t}-\tilde{\theta }∥}_{2}^{2}\right\}\\ \;=\tilde{\theta }-\frac{\overset{K}{\underset{k=1}{\sum }}{∥{\hat{g}}_{t}^{k}-{\hat{g}}_{t-1}^{k}∥}_{2}^{2}}{2} \end{array}$$

**Subproblem** for ${\upsilon_{t}}^{*}$ Given ${\hat{G}}_{t}$,θ_*t*_,***H***_*t*_,***Ŝ***_*t*_, $\upsilon_{t}^{k}$ can be optimized with the following equation.


(11) $$\begin{array}{l} \upsilon_{t}^{k*}=\underset{\upsilon_{t}^{k}}{\arg\min}\frac{1}{2}\overset{K}{\underset{k=1}{\sum }}{∥\upsilon_{t}^{k}\tilde{u}⊙h_{t}^{k}∥}_{2}^{2}+\frac{\lambda_{1}}{2}\overset{K}{\underset{k=1}{\sum }}{∥\upsilon_{t}^{k}-\upsilon_{0}^{k}∥}_{2}^{2}\\ \;=\frac{\lambda_{1}\upsilon_{0}^{k}}{{\left(\tilde{u}⊙h_{t}^{k}\right)}^{⊤}\left(\tilde{u}⊙h_{t}^{k}\right)+\lambda_{1}} \end{array}$$

**Lagrangian multiplier updating**:


(12) $$\begin{array}{l} {\hat{S}}_{t}^{i+1}={\hat{S}}_{t}{}^{i}+\mu^{i}({\hat{G}}_{t}{}^{i+1}-{\hat{H}}_{t}{}^{i+1}) \end{array}$$

Where, *i* and *i* + 1 represent the previous and current iterations. The new ***Ĝ***_*t*_ ***Ĥ*** obtained from the above optimization solution is used to update the Lagrangian multiplier. The regularization constant observes the updating laws of $\mu^{i+1}=min\left(\mu_{max},\beta \mu^{i}\right)$, thereby ensuring the convergence of the integral model according to ADMM.

### 2.4. Historical model retrieval module

Most original tracking methods use linear interpolation with a constant learning rate β, like Equation 13, to update the filter. However, such an updating method not only causes the tracker to indiscriminately treat all the historical information but also results in filter pollution and degradation. The tracking result is poor when faced with complex scenes, such as partial occlusion, and camera defocus. Too high a learning rate causes the tracker to easily overfit and then neglect historical information, while too low a learning rate disenables the tracker from effectively learning the change of targets. Considering that the human brain can recall historical memory to make the best choice when identifying targets and HMTS tracker, the history filter, namely the historical model retrieval module is retrieved, and the best filter of the current frame is obtained by selecting and linear interpolating several effective filters. Specifically, historical filters are saved first, and a filter library is built. After the training phase of each frame, the correlation between each template and the current sample image is calculated. Several historical templates with the highest scores are selected and the scores are used as weights to linear interpolate them, thereby obtaining a tracking template for the next frame object location. This module is described below in detail with mathematical symbols.


(13) $$\begin{array}{l} h_{t}=\beta h+\left(1-\beta \right)h_{t-1} \end{array}$$

Similiar to HMTS tracker (Chen et al., 2022), this method retains the filter for each frame as the historical model ***H***_*hist*_. However, the HMTS tracker builds the filters library with all historical filters, which causes much computing burden and redundancy. For example, when tracking to the end of a long video, there are numerous historical filters, and there is great similarity in target appearance between the current filter and the front filter. Therefore, the size is fixed to ϕ_*hist*_ and the filters library is constructed as $H_{hist}={\left\{\left(h_{i},s_{i}\right)\right\}}_{i=1}^{\phi_{hist}}$. *s*_*i*_ refers to the score of each historical model.

As expressed by the regression term in the loss function Equation 3, the convolution results of the trained filter and sample should ideally present a Gaussian shape centered on the target, namely the label ***y***. The basis of correlation filtering theory is as below: the more similar the two signals are, the greater the correlation between them is. Thus, like the HMTS tracker, the *s*_*i*_ is defined as the correlation between the label ***y*** and the convolution results ***R***_*i*_ of different historical filters ***H***_*i*_, *i* ∈ [1, ϕ_*hist*_] and the current frame target samples ***X***_*t*_. The equation of *s*_*i*_ is as follows:


(14) $$\begin{array}{l} \;s_{i}=max\left(F^{-1}\left(y^{H}R_{i}\right)\right)\\ R_{i}={∥\overset{K}{\underset{k=1}{\sum }}x_{t}^{k}⊛h_{i}^{k}∥}_{2}^{2} \end{array}$$

Where $F^{-1}$ represents the inverse Fourier transform,^*H*^ indicates the conjugate transpose, and *max*(·) refers to the maximum of the vector.

After the tracker training phase in accordance with Section 2.3, Equation (14) is adopted to calculate the scores of the trained filter in the current frame and historical filters in the filters library. Next, the historical model with the lowest score in the filter library is replaced by the filter trained from the current frame, thereby ensuring no change in the number of filters in the library. It needs to be noted that, since the first frame is the most accurate manually labeled target information, the filter of the first frame shall always remain in the filter library. The filter ***h***_*t*_ used for object detection in the next frame can be obtained by a linear weighting of the filters with the top ϕ_*scores*_ scores.


(15) $$\begin{array}{r} h_{t}=\underset{i}{\sum }s_{i}h_{i}\\ s.t.Rank\left(s_{i}\right)\geq \phi_{scores} \end{array}$$

Where, *Rank*(*s*_*i*_) represents the index of *s*_*i*_ in the set ${\left\{s_{i}\right\}}_{i=1}^{\phi_{hist}}$, which is ranked in descending and *i* ∈ [1, ϕ_*hist*_]. It needs to be noted that the filter trained in the first frame always participates in the calculation of Equation 15 and it is given the lowest weight in ϕ_*scores*_ filters if *Rank*(*s*_1_) ≤ ϕ_*scores*_.

## 3. Experiments

In this section, the tracking performance of the proposed tracker is evaluated against the nine state-of-the-art trackers, namely AutoTrack, ASRCF, ECO-HC, STRCF, SRDCF, BACF, LADCF (Xu et al., 2019), MCCT-H (Wang et al., 2018) and Staple (Bertinetto et al., 2016a) on two difficult UAV benchmarks (UAV123 Mueller et al., 2016 and VisDrone2018-test-dev Zhu et al., 2018). For the measurement of the performance of the aforementioned trackers, the employed evaluation metric named one-pass evaluation(OPE) contained two indicators, namely Precision Rate and Success Rate. It needs to be noted that the precision plot threshold is set to 10 pixels in UAV123 and to 21 pixels in VisDrone2018-test-dev, when considering the different target sizes from different UAV datasets.

### 3.1. Implementation details

Our tracker was used in MATLAB-2017a with an Intel i7-9750H CPU, and 16GB of RAM, and runs at a 25 FPS average with hand-crafted characteristics for target representation. The common hyper-parameters are kept to the same values as the baseline Autotrack, namely δ = 0.2, ν = 2 × 10^−5^, and ζ = 13. The SR constraint coefficient λ_1_ and the response aberrance regularization constraint coefficient λ_2_ which are unique to the proposed tracker, are set as 0.71 and 0.001, respectively. In the historical model retrieval module, ϕ_*hist*_ = 30 and ϕ_*scores*_ = 20 are determined. As for the ADMM algorithm, the number of iterations is set as 4, β = 10, and $\mu_{max}=10^{4}$, which also shares the same parameters as in Autotrack.

### 3.2. Quantitative evaluation

**Figure 1:**
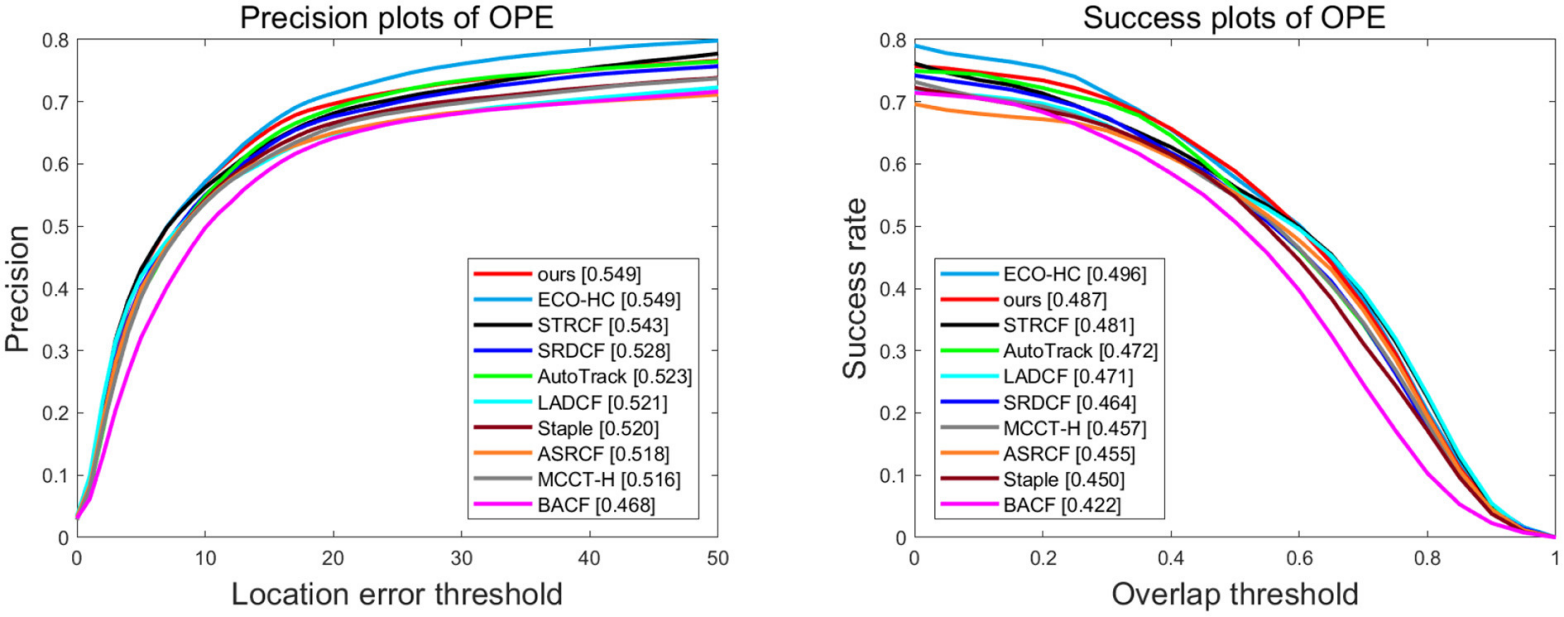
Overall performance of ten trackers on UAV123. The legend of success plot represents the area under the curve score of each tracker. The legend of the precision plot refers to the threshold scores at 10 pixels.

**UAV123** is the most commonly used dataset in UAV object tracking, with 123 videos with more than 110K frames composed. In these sequences, 12 of the challenging attributes involved, such as background clutter, aspect ratio change, and similar object, required a more accurate and stable tracking algorithm. The quantitative comparison of different trackers is shown in Figure 1, and it can be observed that our tracker shows the best precision with the second success rate, slightly lower than ECO-HC. However, the proposed method achieves a remarkable advantage of 2.6% in precision and 1.5% in success rate, compared with the baseline tracker Autotrack.

**Figure 2:**
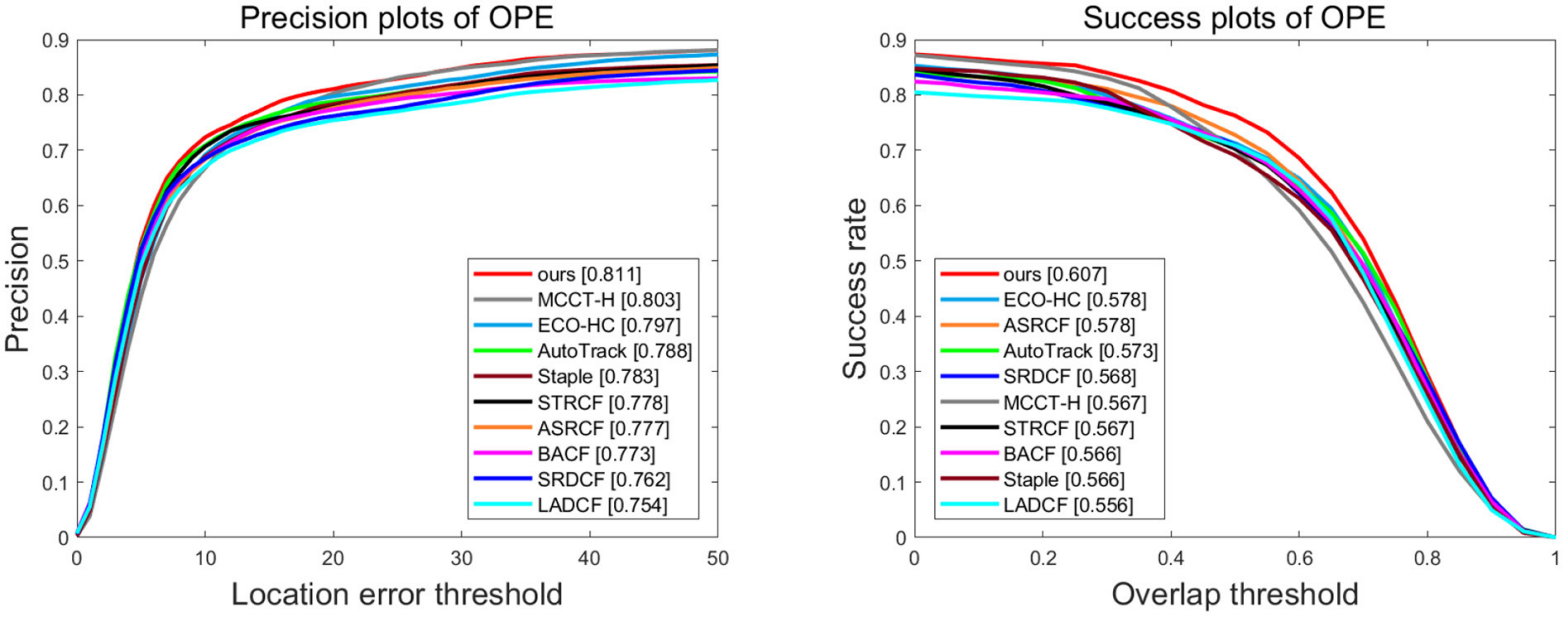
Overall performance of ten trackers on Visdrone2018-test-dev. The legend of success plot represents the area under the curve score of each tracker. The legend of the precision plot refers to threshold scores at 21 pixels.

**VisDrone2018-test-dev** is a dataset that is especially proposed for aerial object tracking competition. It consists of 35 videos captured from 14 different cities and covers various aspects including such as shooting position, tracking scene, target type, and object density. Different scenarios, weather conditions, and illumination changes are primarily addressed in this dataset. As shown in Figure 2, the proposed tracker is superior to all other evaluated trackers, and it can achieve 81.1% and 60.7% in the distance precision (DP) and the area under the curve (AUC), respectively. By comparing with the baseline tracker, Autotrack, our tracker accomplishes 2.3% and 3.4% of performance gains in precision and success rate, respectively.

### 3.3. Parametric sensitivity

As presented in Section 3.1, some hyper-parameters of the proposed tracker need to be set, namely the spatial-channel regularization constraint coefficient λ_1_ and the response aberrance regularization constraint coefficient λ_2_ in the loss function. In this section, the influence of different configurations on tracking results is investigated. When evaluating each hyper-parameter for a fair comparison, the common parameters are maintained at the same value as in Autotrack and all other parameters are fixed. Considering the operation speed, ϕ_*hist*_ is set as a constant of 30 and ϕ_*scores*_ = 20 is set as a constant of 20 to ensure the efficient use of historical information and the effective reduction of redundancy. Table 1 exhibits the tracking results under different λ_1_,λ_2_ in VisDrone2018-test-dev, where ϕ_*scores*_ is fixed to 20. It can be observed that this tracker yields the best performance with λ_1_ = 0.001 and λ_2_ = 0.71.

**Table 1:** The success rate and precision rate (percentage) related to the varying number of regularization constraint coefficients on VisDrone2018-test-dev.

**Parameter**	λ_**1**_				λ_**2**_			
Value	**0.001**	0.1	0.5	1	**0.71**	0.01	0.1	1
Success Rate	**60.7**	58.4	59.2	59.0	**60.7**	58.5	59.1	59.0
Precision Rate	**81.1**	78.4	79.5	80.2	**81.1**	79.1	80.1	79.5

In historical models, ϕ_*scores*_ = 20 and ϕ_*hist*_ = 30. The threshold of precision rate is set to 21 pixels.

Bold values refer to first place in the experiments.

### 3.4. Ablation experiments

As described in Section 2, in our method loss function is reconstructed by introducing the feature channel weight and aberrance repressed regularization, and an additional historical memory model is added to the baseline Autotrack. To prove the effectiveness of each module, ablation experiments were conducted. The results are shown in Table 2. AutoTrack_csar only reconstructs the loss function, while AutoTrack_hist only adds the historical memory model. As observed, by adding the two modules separately, the performance of the baseline tracker can be improved effectively. Moreover, by joining these two modules simultaneously, our method can achieve excellent performance against the baseline. This is mainly because the fusion of the two enables the tracker to effectively use historical information to prevent background clutter during tracking while establishing a more robust target appearance representation.

**Table 2:** The success rate and precision rate (percentage) of ablation experiments on UAV123.

**Tracker**	**AutoTrack**	**Two regularization**	**Historical memory**	**Precision rate**	**Success rate**
AutoTrack	✓			52.3	47.2
AutoTrack_csar	✓	✓		53.7	47.4
AutoTrack_hist	✓		✓	54.2	47.5
Ours	✓	✓	✓	**54.9**	**48.7**

The precision rate threshold is set as 10 pixels.

Bold values refer to first place in the experiments.

### 3.5. Qualitative evaluation

**Figure 3:**
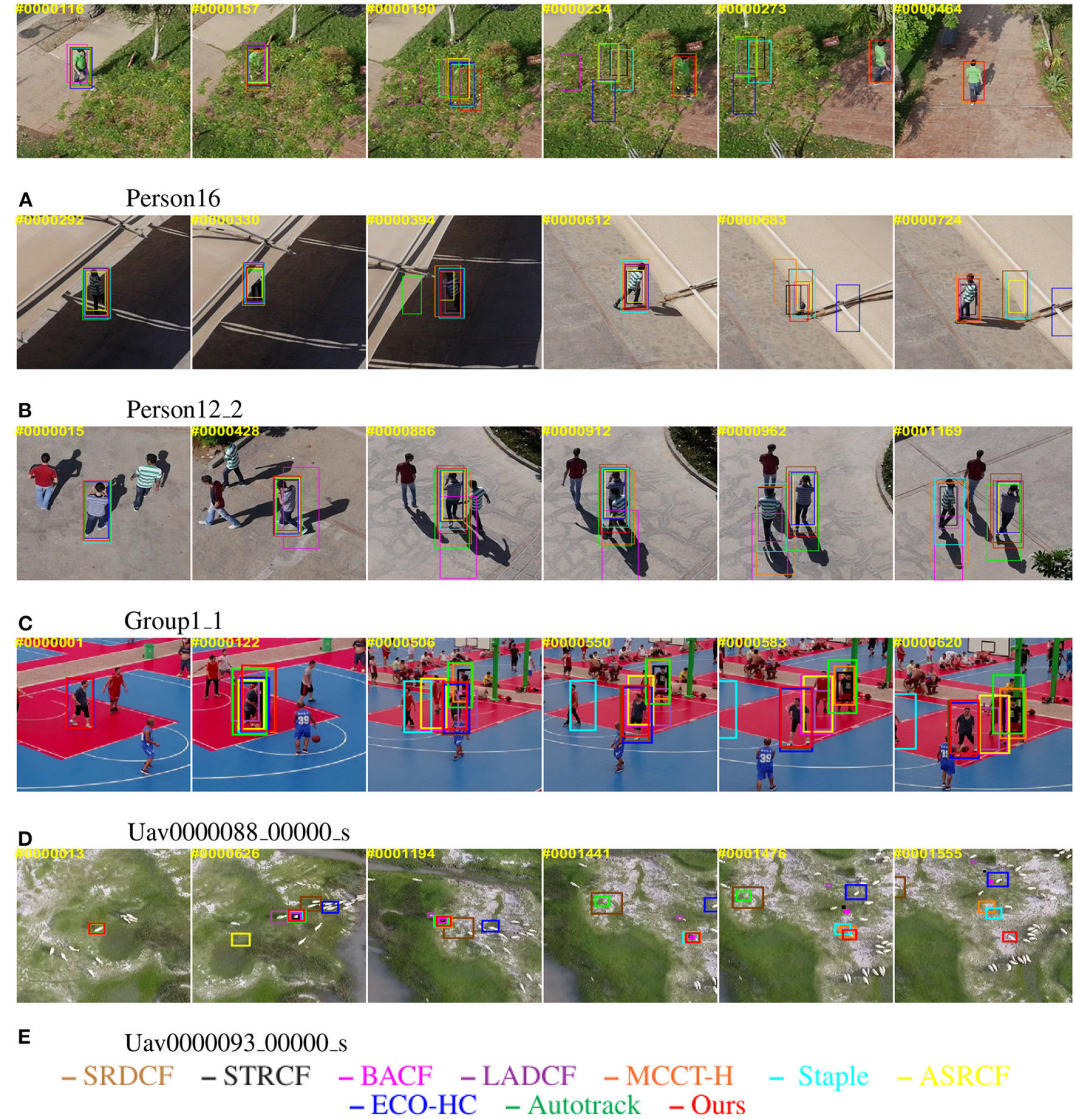
Qualitative performance evaluation of the proposed tracker and the other nine most advanced trackers on the typical UAV videos. The number in the upper left corner refers to the frame number. The tracking boxes in different colors represent the tracking results of different trackers in the frame. **(A)** Person16. **(B)** Person12_2. **(C)** Group1_1. **(D)** Uav0000088_00000_s. **(E)** Uav0000093_00000_s. The photos appearing in this figure have been reused from: ‘A benchmark and simulator for uav tracking’ and ‘Vision meets drones: a challenge’. The corresponding website are ‘https://cemse.kaust.edu.sa/ivul/uav123’ and ‘http://aiskyeye.com’.

In this subsection, the qualitative comparison is given to the proposed method and the aforementioned 9 state-of-the-art algorithms to better demonstrate the performance of each tracker in Figure 3. The above image sequences (containing person16, person12_2, group1_1 in UAV123 and Uav0000088_00000_s, and Uav0000093_00000_s in VisDrone2018-test-dev) mainly include three challenging attributes, namely similar object (SOB), background clutters (BC), and occlusion (OC). It can be observed that our tracker is effective in solving these difficult issues, and can locate the targets accurately.

When facing a similar object and background clutter, aberrance repressed regularization can help the tracker in accurately perceiving and fully restraining the interference regions in advance. Simultaneously, dynamic feature channel weight realizes the independent filtering of different dimensional features, thereby encouraging the filters to focus on more reliable and discriminative features between the target and a cluttered background. By jointly modeling the above two constraints, the tracker can learn the robust features of the target according to the environment and the interference from a cluttered background.

When there is an occlusion, the trackers can learn the features of the block and lose the target information, thus leading to model drift and a failure of tracking. With the introduction of a historical model retrieval module in our method, the tracker has a memory function similar to the human brain by saving a history template. The method of dynamic updating of the template encourages the tracker to reduce the learning rate when the training sample is abnormal, thereby effectively reducing the probability of template pollution. The memory function of the tracker also guarantees that the method can accurately lock the target again after the disappearance of the occlusion.

In summary, when challenging attributes occur during tracking, the addition of the two constraints endows the tracker with the ability to select the most distinguishing feature for sensing and suppressing the interference around the target, while the historical model retrieval module effectively reduces the pollution of interference and noise to the tracker. However, when meeting viewpoint change and rotation, the performance of our tracker is reduced because of rapid changes in the appearance of the target. In the future, we will explore how to refine tracking results to solve such problems.

## 4. Conclusion

Based on the idea that the brain can perceive interference information in the background, select the optimal features independently to describe the target, and use historical memory to achieve accurate target location in the current frame, in this paper, we propose a UAV tracking algorithm on the basis of repressed dynamic aberrance and a channel selective correlation filter with a historical model retrieval module combined. By jointly modeling feature channel weight and the aberrance repressed regularization, our tracker could restrain the potential distractors, and highlight the valuable features in the channel domain, thereby constructing a robust target appearance. With a historical model retrieval module, our tracker can make full use of the historical information to update the tracker, while effectively avoiding tracking drift. The experimental results on the two public UAV benchmarks demonstrate that the proposed method achieves better tracking results than the other advanced algorithms.

## Data availability statement

The original contributions presented in the study are included in the article/supplementary material, further inquiries can be directed to the corresponding author.

## Author contributions

JC and JW proposed the basic idea of this method and wrote the code together. JC completed theoretical modeling. JC and LZ performed the experiments and data analysis. JC wrote the first draft of the manuscript. JW and LZ revised the manuscript. All authors contributed to the article and approved the submitted version.
